# Identification of New Key Genes and Their Association with Breast Cancer Occurrence and Poor Survival Using In Silico and In Vitro Methods

**DOI:** 10.3390/biomedicines11051271

**Published:** 2023-04-25

**Authors:** Rafat Ali, Armiya Sultan, Romana Ishrat, Shafiul Haque, Nida Jamil Khan, Miguel Angel Prieto

**Affiliations:** 1 Department of Biosciences, Jamia Millia Islamia (A Central University), New Delhi 110025, India; 2 Center for Interdisciplinary Research in Basic Sciences, Jamia Millia Islamia (A Central University), New Delhi 110025, India; 3 Research and Scientific Studies Unit, College of Nursing and Allied Health Sciences, Jazan University, Jazan 45142, Saudi Arabia; 4 Gilbert and Rose-Marie Chagoury School of Medicine, Lebanese American University, Beirut P.O. Box 36, Lebanon; 5 Centre of Medical and Bio-Allied Health Sciences Research, Ajman University, Ajman P.O. Box 346, United Arab Emirates; 6 Nutrition and Bromatology Group, Department of Analytical Chemistry and Food Science, Faculty of Science, Universidade de Vigo, E32004 Ourense, Spain

**Keywords:** breast cancer, differentially expressed genes, down regulated genes, poor survival, up regulated genes

## Abstract

Breast cancer is one of the most prevalent types of cancer diagnosed globally and continues to have a significant impact on the global number of cancer deaths. Despite all efforts of epidemiological and experimental research, therapeutic concepts in cancer are still unsatisfactory. Gene expression datasets are widely used to discover the new biomarkers and molecular therapeutic targets in diseases. In the present study, we analyzed four datasets using R packages with accession number GSE29044, GSE42568, GSE89116, and GSE109169 retrieved from NCBI-GEO and differential expressed genes (DEGs) were identified. Protein–protein interaction (PPI) network was constructed to screen the key genes. Subsequently, the GO function and KEGG pathways were analyzed to determine the biological function of key genes. Expression profile of key genes was validated in MCF-7 and MDA-MB-231 human breast cancer cell lines using qRT-PCR. Overall expression level and stage wise expression pattern of key genes was determined by GEPIA. The bc-GenExMiner was used to compare expression level of genes among groups of patients with respect to age factor. OncoLnc was used to analyze the effect of expression levels of *LAMA2*, *TIMP4*, and *TMTC1* on the survival of breast cancer patients. We identified nine key genes, of which COL11A1, MMP11, and COL10A1 were found up-regulated and PCOLCE2, *LAMA2*, *TMTC1*, ADAMTS5, *TIMP4*, and RSPO3 were found down-regulated. Similar expression pattern of seven among nine genes (except ADAMTS5 and RSPO3) was observed in MCF-7 and MDA-MB-231 cells. Further, we found that *LAMA2*, *TMTC1*, and *TIMP4* were significantly expressed among different age groups of patients. *LAMA2* and *TIMP4* were found significantly associated and *TMTC1* was found less correlated with breast cancer occurrence. We found that the expression level of *LAMA2*, *TIMP4*, and *TMTC1* was abnormal in all TCGA tumors and significantly associated with poor survival.

## 1. Introduction

Breast cancer is one of the most prevalent types of cancer diagnosed globally. Its burden has been rising over the past decades accounting for 1 in 8 cancer diagnoses worldwide and a total of 2.3 million new cases in both sexes combined [1,2]. Breast cancer continues to have a significant impact on the global number of cancer deaths. Estimation reflected that about 685,000 women died from breast cancer in 2020, corresponding to 16% or 1 in every 6 cancer deaths in women [1]. Breast cancer cases are predicted to reach 4.4 million in 2070 [3]. The incidence of breast cancer varies around the world, with the highest rates typically found in more developed countries [4,5]. However, the number of breast cancer cases is also exponentially increasing in low- and middle-income countries [6]. Breast cancer incidence is strongly linked to human development and is higher among nations with the highest human development index [2]. In females, the worldwide age-standardized incidence rate is estimated to be 48/100,000, ranging from less than 30/100,000 in Sub-Saharan Africa to more than 70/100,000 in Western Europe and North America [7]. Although the relative incidence of breast cancer is highest in the most developed parts of the world, since less developed regions have much bigger populations means more than half of all breast cancer cases are identified in low- and middle-income nations, creating a significant considerable disease burden [2,7]. Despite the high incidence of the disease, early detection and improved treatment options have led to a decrease in breast cancer deaths in many countries. However, access to early detection and treatment options remains a concern in low- and middle-income countries, and more efforts are needed to address this issue.

The reported worldwide differences in the incidence of breast cancer must be viewed in the context of the disease’s recognized risk factors. There are several risk factors that have been associated with an increased risk of developing breast cancer. Some of the most well-known major risk factors include older age, breast density and family history of breast cancer, early age menarche, late age at first full-term pregnancy, shorter breastfeeding periods, use of hormonal menopausal therapy, use of oral contraceptives, high fat dietary, high body mass index, physical inactivity, obesity and exposure to tobacco [4,8,9]. Highest incidence rate of breast cancer in developed countries is likely due to a combination of factors such as an aging population, lifestyle factors, and improved access to healthcare and cancer screening programs [10]). Increasing incidence of breast cancer in low- and middle-income countries is likely attributed to changes in lifestyle and reproductive patterns, such as an increase in the use of hormone replacement therapy (HRT) and a decrease in the number of children born to women [4]. Additionally, increased urbanization and the adoption of western lifestyles in these countries may also contribute to the rise in breast cancer incidence [6]. It’s worth noting that having one or more of these risk factors does not mean that a woman will develop breast cancer, and many women who develop the disease do not have any known risk factors.

Early diagnosis and treatment are crucial for a positive outcome of any disease. The treatment of breast cancer depends on several factors, including the stage and type of cancer, as well as the patient’s overall health. Despite modern advances in target therapy method, the result of treating breast cancer is still unsatisfactory. Thus, understanding the molecular mechanisms of breast cancer progression and identifying novel potential prognostic biomarkers and molecular targets are urgently needed. This will also give deep insight for the diagnosis and treatment at every stage of breast cancer. Currently, in-silico techniques followed by in-vitro or in-vivo validations are widely used to identify the key regulatory genes and to determine their structural and relational aspects with disease [11]. This approach offers an ideal way to screen large gene expression profiles of normal and clinical populations to understand the genomic mechanisms contributing for the development and progression of different diseases [12]. Nowadays, high-throughput microarray technology and bioinformatics analysis is widely used to find gene expression variations between malignant and non-cancerous tissues, assess differentially expressed genes (DEGs), and uncover the pathways that contribute to carcinogenesis and cancer development. New biomarkers and therapeutic targets have been discovered from cancer-associated gene expression profiles which showed reliable outcome in clinical studies [13,14,15,16].

In the present study, NCBI-GEO database was accessed to retrieve four cancer-associated gene expression profiles. These datasets were analyzed by integrated in-silico methods to determine the DEGs associated with breast cancer. Key regulatory genes having high correlation with sample clinical characteristics were extracted and further validated using MCF-7 and MDA-MB-231 human breast cancer cell lines.

## 2. Materials and Methods

### 2.1. Retrieval of Datsets and Extraction of Differentially Expressed Genes (DEGs)

The NCBI-GEO (http://www.ncbi.nlm.nih.gov/geo/ (accessed on 3 March 2021)) database is a free public database of microarrays. It is used for gene expression datasets and platform records. Four datasets with accession number GSE29044, GSE42568, GSE89116, and GSE109169 were retrieved from the GEO database (https://www.ncbi.nlm.nih.gov/geo/ (accessed on 3 March 2021)) and analyzed using R packages. DEGs between the cancer and normal samples were identified by applying statistical parameters. The DEGs with FC ≥ 1.5 for up regulated and FC ≤ −1.5 for down regulated and adjust *p* < 0.05 were considered as the cut-off criteria.

### 2.2. Construction of Protein-Protein Interaction (PPI) Network

The identified DEGs were further used as input in network building. The PPI network was constructed by using the STRING database. A total of 161 DEGs extracted from all the four datasets were used to make the PPI network. Further, analysis of the network and their visualization process was performed by Cytoscape software (version 3.7.1) [17]. 

### 2.3. Characterization of Networks Topological Properties

Topological parameter behaviors were explored to determine the structural properties of complex networks by using the Network Analyser plugin in Cytoscape [18]. The Topological properties analyzed are explained below:

### 2.4. Probability of Degree Distribution

The degree distribution of a PPI network is a probability distribution of a node to have degree k. The ‘k’ represents the number of links of a node that connects with other nodes. For example, If G = (N, E) describes a graph of a network, where N and E represent the node and edges of the network respectively. The, the degree distribution probability (P (k)) of a network is measured by the equation [19,20]:

P (k) = nk/N(1)

where, nk = number of nodes having degree k and N = total number of nodes in the network. 

### 2.5. Betweenness Centrality

In a node of a PPI network, Betweenness centrality characterizes the prominence of information that is flowing through one node to another by following a shortest path [21,22]. The geodesic paths are shown from node i to node j by ’dij (v)’ which passes through node ’v’ and ’dij’. The Betweenness centrality of a node v can be measured by the following equation:

CB (v) = ∑I,j,I ≠ j ≠ k (dij(v)/dij)(2)

### 2.6. Closeness Centrality

Closeness centrality characterizes how quickly the information is travelling through the network i.e., from one node of the PPI network to another node [23]. The Closeness centrality of the node i is described as the reciprocal average length of the geodesic paths between the node and all other nodes connected to it in the network. Closeness Centrality is measured by the following equation:

CC (k) =n/(∑_j d_ij)(3)

where, dij in a PPI network presents length of the geodesic path between nodes i and j. n presents total number of nodes in the PPI network connected to node i.

### 2.7. Community Detection: Leading Eigen Vector Approach

To characterize the modular nature, attributes, and organizing principle of the hierarchical network, the activities of the created network were defined at various levels of hierarchy [24]. In this work, the Leading Eigen Vector technique (LEV) [25,26] from the package ‘igraph’ [27] was utilized in R to discover communities. The LEV technique is the most promising for community discovery since it computes the Eigen value for each connection, demonstrating the significance of each link rather than nodes. We discovered modules from the entire network and then sub-modules from the modules at each level of organisation to retrieve just the theme (Figure S1).

### 2.8. Genes Tracing across the Networks

Identifying the primary forces that influence the regulation of the PPI network is one special problem [28]. This was accomplished by gene tracing using the LCV method in CYTOSCAPE. This gene tracing was done up to the motif level in several modules/sub-modules derived through clustering. The network’s regulator was identified by tracking the most significant and influential nodes within the network’s construction.

### 2.9. Gene Ontology and Pathway Analysis of Key Genes 

The DAVID (https://david.ncifcrf.gov/ (accessed on 7 June 2021)) and KEGG databases were utilized for GO and pathway analysis to explore the function and associated pathways of the key genes [29,30]. Gene ontology (GO) analysis annotates genes and gene products using functions such as molecular function, biological pathways, and cellular components [31]. KEGG is a collection of genomic and enzymatic techniques, as well as an online library of biological chemical energy [32]. KEGG is a resource for comprehensive gene function analysis as well as associated high-level genome functional information. DAVID can give full biological function annotation information for high-throughput gene expression [33]. As a result, we used DAVID online tools to perform GO and KEGG pathway analyses on the key genes at the functional level. A *p* < 0.05 was considered statistically significantly different. 

### 2.10. GEPIA Analysis

GEPIA (http://gepia.cancer-pku.cn/detail.php (accessed on 7 June 2021)) is a specialized web server for the analysis of RNA-seq data of 9736 tumors and 8587 normal samples from the TCGA (http://portal.gdc.cancer.gov/ (accessed on 7 June 2021)) and the GTEx (http://gtexportal.org/home/ (accessed on 7 June 2021)) projects [34]. Using the GEPIA web server, expression level, survival, and expression level at different stages of key genes was studied. The predictive value of all key genes was assessed throughout the TCGA dataset using the GEPIA web server’s default parameters. The default values for all parameters were used, and the cut-off value was set at median = 50 percent. *p* < 0.05 was used to indicate a statistically significant difference in the HR.

### 2.11. bc-GenExMiner Analysis

The Breast Cancer Gene Expression Miner v4.4, (http://bcgenex.ico.unicancer.fr/BC-GEM/GEM-Accueil.php?js=1 (accessed on 7 June 2021)) a DNA microarray and RNA-seq database may be used to look at gene expression and predict prognosis. We looked at the relationship between gene expression of the key genes and clinic pathological parameters like age, and specific region of breast cancer patient sample using microarray data. Furthermore, we conducted a prognostic analysis of the genes as well.

### 2.12. UALCAN Analysis

The online cancer transcriptome database UALCAN (http://ualcan.path.uab.edu/ (accessed on 7 June 2021)) is meant to enable simple access to publicly accessible cancer transcriptome data (TCGA and MET500 transcriptome sequencing) [35]. UALCAN is a comprehensive, user-friendly, and interactive web resource for analyzing cancer OMICS data. UALCAN enables researchers to access Level 3 RNA-seq data from The Cancer Genome Atlas (TCGA) and perform gene expression and survival analysis on about 20,500 protein-coding genes in 33 different tumor types [35]. It’s written in PERL-CGI and has high-resolution visuals created using JavaScript and CSS. The Clinical Proteomic Tumor Analysis Consortium (CPTAC) Confirmatory/Discovery dataset is now available in UALCAN for protein expression analysis. The level of expression of key genes in normal breast tissue and primary invasive breast cancer was compared using this database.

### 2.13. OncoLnc Analysis

#### 2.13.1. Survival Analysis

An overall survival analysis for patients with breast cancer was performed using the OncoLnc program (www.oncolnc.org (accessed on 7 June 2021)). OncoLnc is an interactive online application that allows users to explore the survival data of 8647 individuals from 21 cancer studies in The Cancer Genome Atlas (TCGA), as well as TCGA’s mRNA and miRNA RNA-Seq expression data. The software allows you to create Kaplan-Meier graphs that are stratified by gene expression levels. In survival analysis, log-rank *p*-values were collected. High and low groups were defined as the 80th (upper) and 20th (lower) percentiles, respectively. On 10 November 2021, the survival rate curves were produced using OncoLnc (http://www.OncoLnc.org/ (accessed on 7 June 2021)) [36]. The upper and lower quartiles were used to divide the high and low expression groups. 

#### 2.13.2. Cell Lines, Culture and Validation of Key Regulatory Genes by qRT-PCR 

The in-vitro validation of the key regulatory genes was done by using human breast cancer cell lines namely MCF-7 and MDA-MB-231. Cells were procured from National Center for Cell Science (NCCS), Pune, India. Cells were grown and maintained in DMEM media supplemented with 10% FBS and 1% penicillin/streptomycin salt solution at 37 °C in a humidified 5% CO_2_ incubator. RNA was extracted from 70 to 75% confluent cells using a Trizol reagent (Ambion, Carlsbad, CA, USA). RNA was quantified using nanodrop, and 1000 ng of RNA was reverse transcribed into cDNA using Verso cDNA Synthesis Kit (Thermo Fisher Scientific, Waltham, MA, USA). qRT-PCR was conducted using SYBR™ Green Master Mix (Thermo Fisher Scientific, USA) and The Applied Biosystems^®^ QuantStudio™ 6 Flex Real-Time PCR System to determine the expression profile of nine key regulatory genes obtained through bioinformatics analysis. 18s was used as an endogenous control to normalize the target genes. Thereafter, relative fold change in expression level was calculated for all the nine key regulatory genes. Primer list of respective genes is presented in Table S1.

## 3. Results

### 3.1. Characteristics of Datasets Used to Extract Common DEGs

Four datasets having accession number GSE29044, GSE42568, GSE89116, and GSE109169 were obtained from freely accessible NCBI-GEO database. Detailed information of the datasets is presented in Table 1. GSE29044 dataset was based on the GPL570 platform containing 6 samples of early tumour patients and 5 samples of early normal aged between 20–35 years old and 25 samples of late tumour and 7 samples of late normal whose age was greater than 55 years old. GSE89116 dataset was based on the GPL6947 platform containing 11 samples of early tumour patients (max. age 38 years) and 4 samples of early normal (max. age 35 years) and 13 samples of late tumour (max. age 80 years) and 5 samples of late normal (max. age 80 years). GSE109169 dataset was based on the GPL570 platform containing 5 samples of early tumour patients and 5 samples of early normal aged less than 40 years and 20 samples of late tumour and 20 samples of late normal aged more than 40 years. GSE42568 dataset was based on the GPL570 platform containing 104 tumour samples of patients aged between 31 to 89 years old at the time of diagnosis and 17 normal samples with no defined age. 

The microarray expression profiles are widely utilized to study the gene expression on a genome-wide scale. There are few algorithms available that are used to correct the batch effects before analysing the microarray data. We employed the Empirical Bayes method built-in function in LIMMA, in combination with the fit2 function. Effective in-silico methods are required for the integration of Meta-analyses-based microarray data. These in silico methods are used to merge efficiently various microarray datasets without considering the impact of demographics, experimental designs, and specimen sources [19].

### 3.2. Identification of Common DEGs

**Figure 1:**
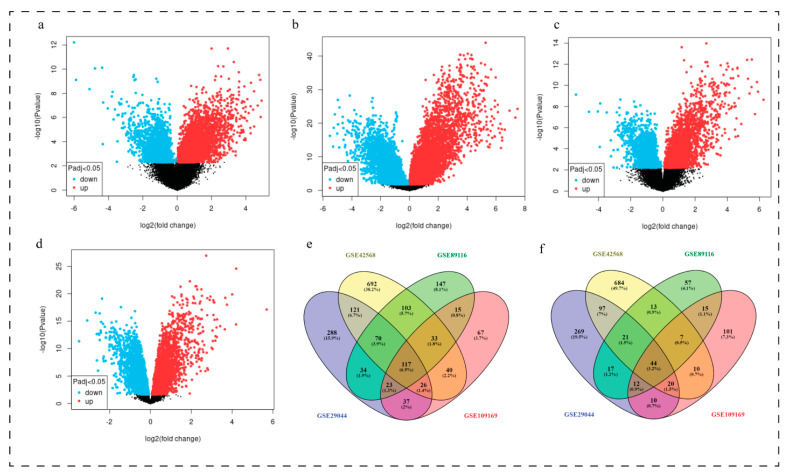
DEGs in breast cancer tissues. Volcano plot presenting DEGs in breast cancer tissues in datasets. (**a**) GSE29044 (**b**) GSE42568 (**c**) GSE89116 (**d**) GSE109169 (**e**)Venn diagram represented the down regulated overlapping DEGs among GSE29044, GSE42568, GSE89116, and GSE109169 datasets. (**f**) Venn diagram represented the up-regulated overlapping DEGs among GSE29044, GSE42568, GSE89116, and GSE109169 datasets.

Only those DEGs that surpassed the cut-off criteria of FC ≥ 1.5 for up re-gulated genes and FC ≤ −1.5 for down regulated genes and adjust *p* < 0.05 in all the four data series were considered as the significant DEGs Figure 1. Total 161 common DEGs were extracted from all the four datasets by Venn diagram Figure 1e,f. Among these 161 common DEGs, 44 genes were up regulated and 117 genes were down regulated. List of all the common DEGs is presented in Table 2. Volcano plot represented DEGs in breast cancer tissues and non-tumor samples in datasets. (a) GSE29044 (b) GSE42568 (c) GSE89116 (d) GSE109169 (e) Venn diagram represented the down regulated overlapping DEGs among GSE29044, GSE42568, GSE89116, and GSE109169 datasets. (f) Venn diagram represented the up-regulated overlapping DEGs among GSE29044, GSE42568, GSE89116, and GSE109169 datasets.

### 3.3. Protein-Protein Interaction (PPI) Network of DEGs

**Figure 2:**
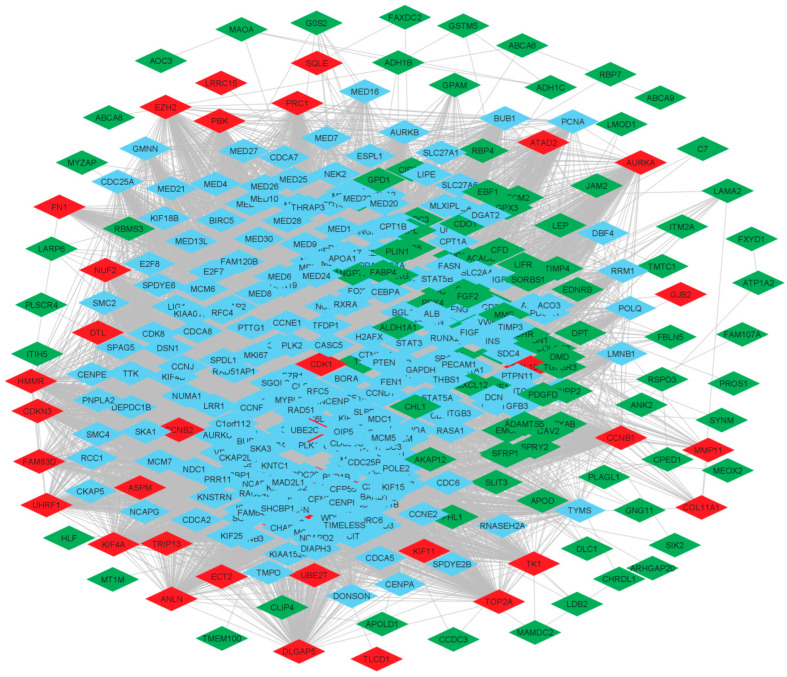
Protein-protein interaction network showing down-regulated (green) DEGs and up-regulated (red) DEGs. Blue colour is presenting supporting genes.

The PPI network was constructed by using the String online database and was imported into Cytoscape v. 3.80, which supports the visualization of bipartite graph of gene-gene linking/interaction/regulation, reflecting gene-disease associations. This also provides gene-centric views of the network data [20]. The Probe Ids of common DEGs were mapped to their corresponding gene symbols to create the native network Figure 2. The PPI network showed 449 nodes and 18214 edges. The network was characterized by several properties such as average number of neighbours was found 80.811, network diameter was 7, characteristics path length was 2.228, clustering coefficient was 0.662, network density was 0.180, network heterogeneity was 0.783, network centralization was 0.375, connected component was 1 and analysis time of 0.484 s. 

### 3.4. Community Detection by Leading Eigen Vector Method

**Figure 3:**
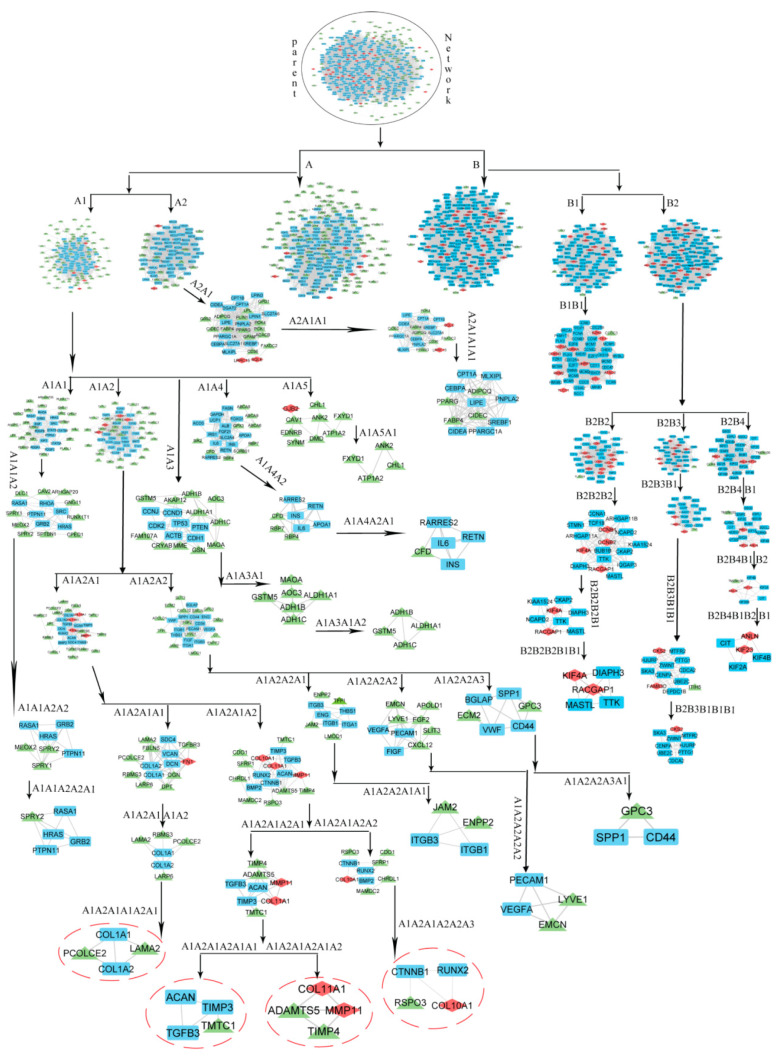
Network/modules/sub-modules at different levels of network.

To characterise the modular nature, attributes, and organising principle of the hierarchical network, the activities of the created network were defined at various levels of hierarchy. For this, Leading Eigen Vector (LEV) method from the package ‘igraph’ was utilised in R to discover communities. The LEV technique is the most promising for community discovery since it computes the Eigen value for each connection, demonstrating the significance of each link rather than nodes. We discovered modules from the entire network and then sub-modules from the modules at each level of organisation to retrieve just theme (Figure 3). 

The modules from the native network along with sub-modules from modules at each level of organization were identified until only motifs remained i.e., unbreakable part of the network.

### 3.5. Identification of Key Regulators and Properties of Breast Cancer Network

In the constructed PPI Network, we found two communities that were further broken down into sub-community and sub-sub-communities up to seventh level. The analysis of modular structure along its arrangement was carried out by the Newman and Girvan standard community finding techniques [37]. These techniques were employed at different organizational levels (Figure 3). We found that our PPI network is organized hierarchically through seven different levels. 

The leading hubs (nodes) are considered as essential regulators based on changes in the activities of proteins/genes along with their regulating mechanisms. However, all the leading hubs cannot be considered as key regulators for the progression of disease. Only those that regulate the network from top to bottom (where the PPI network cannot be further divided into sub-community and form motif) are considered as important leading hubs. These leading hubs are termed as Key Regulators (KRs). Because these KRs are deeply rooted reaching to motif level (fundamental regulating unit) through different community or sub-community levels of the organization in the PPI network. KRs act as the backbone for network’s stability and capacitate it to tackle any unacceptable changes. Topological properties of any PPI network assist to gain deep insight of a network, its behaviour, somehow function, characteristics, and how and what the network is [38]. Therefore, we described some topological properties namely Betweenness Centrality, Degree Centrality, and Closeness Centrality of our network at almost the last level from the parent network (Figure S2). Betweenness Centrality is a way of detecting the amount of influence a node has over the flow of information in a graph. It is often used to find nodes that serve as a bridge from one part of a graph to another. The measure of Degree Centrality presents popular nodes within a graph. It measures the number of incoming or outgoing (or both) relationships from a node, depending on the orientation of a relationship projection. Closeness centrality detects nodes that can spread information very efficiently through a graph. The closeness centrality of a node measures its average farness (inverse distance) to all other nodes. Nodes with a high closeness score have the shortest distances to all other nodes [21]. The gene tracing was done up to the motif level in several modules/sub modules derived through clustering. The network’s regulator was identified by tracking the most significant and influential nodes within the network’s construction. Finally, we have found nine key regulators namely *PCOLCE2, LAMA2, TMTC1, ADAMTS5, TIMP4, RSPO3, COL11A1, MMP11, and COL10A1*. 

### 3.6. Gene Ontology and KEGG Pathway Analysis of Key DEGs

All the DEGs were uploaded to the DAVID database (https://david.ncifcrf.gov/ (accessed on 7 June 2021)) for GO analysis. Results showed that the nine key genes were involved in several GO biological processes namely extracellular structure organization, external encapsulating structure organization, extracellular matrix organization, extracellular matrix disassembly, cellular component disassembly, collagen fibril organization, and supramolecular fibre organization (Table S2). Results showed GO Cellular Components associated with key genes include endoplasmic reticulum lumen, intracellular organelle lumen, collagen-containing extracellular matrix, basement membrane, and Golgi lumen (Table S3). GO Molecular Function analysis showed the key genes were associated with the molecular functions namely metalloendopeptidase activity, metallopeptidase activity, metalloendopeptidase inhibitor activity, and endopeptidase activity (Table S4). Results showed that the key genes were enriched in protein digestion and absorption and viral myocarditis according to the KEGG pathway analysis (Table S5).

### 3.7. Gene Expression Profiling of Key DEGs

**Figure 4:**
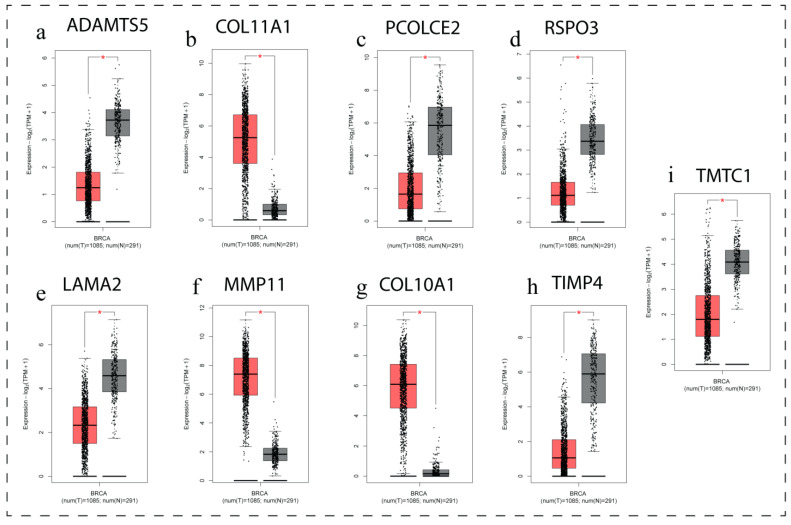
Comparisons of the expression of the nine genes between breast cancer and normal breast tissues in TCGA and GTEx based on GEPIA. The Y axis represents the log2 (TPM + 1) for gene expression. The Gray bar indicates the normal tissues, and the red bar shows the breast cancer tissues. These figures were derived from GEPIA. TPM: transcripts per kilobase million. The box plots (**a**–**i**) of all nine hub genes demonstrate that the genes were abnormally expressed in breast cancer as compared to normal breast tissue. (**a**) ADAMTS5—down-regulated, (**b**) COL11A1—up-regulated, (**c**) PCOLCE2—down-regulated, (**d**) RSPO3—down-reguloated, (**e**) LAMA2—down-regulated, (**f**) MMP11—up-regulated, (**g**) COL10A1—up-regulated, (**h**) TIMP4—down-regulated, and (**i**) TMTC1—down-regulated. * *p* < 0.05.

GEPIA (Gene Expression Profiling Interactive Analysis) is a web-based tool for analyzing gene expression data. GEPIA was chosen to analyse hub genes, their overall expression level comparison to normal tissues and stage wise expression pattern of breast cancer scenario. The box plot (Figure 4) of all nine hub genes demonstrates that the genes were abnormally expressed in breast cancer as compared to normal breast tissue. The genes namely COL11A1, MMP11 and COL10A1 were found up regulated and PCOLCE2, *LAMA2*, *TMTC1*, ADAMTS5, *TIMP4* and RSPO3 were found down regulated in breast cancer. The details LogFC, *p*-value, and Adj. *p*. Value of all the nine key genes is presented in Table 3.

**Figure 5:**
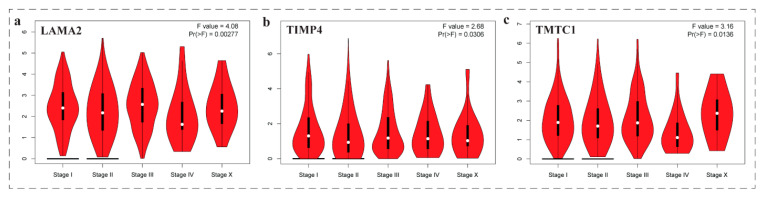
The expression-stage plot of three genes associated with breast cancer. The plots were achieved by the GEPIA web server. The expression-stage plot analysis (violin plots **a**–**c**) revealed that three genes namely (**a**) LAMA2, (**b**) TMTC1, and (**c**) TIMP4 among these nine genes were found significantly associated (*p* < 0.05) with different stages of breast cancer.

The expression-stage plot analysis (violin plots) revealed that three genes namely *LAMA2*, *TMTC1* and *TIMP4* among these nine genes were found significantly associated (*p* < 0.05) with different stages of breast cancer (Figure 5).

### 3.8. Expression Level of Genes among Groups of Patients with Respect to Age Factor

**Figure 6:**
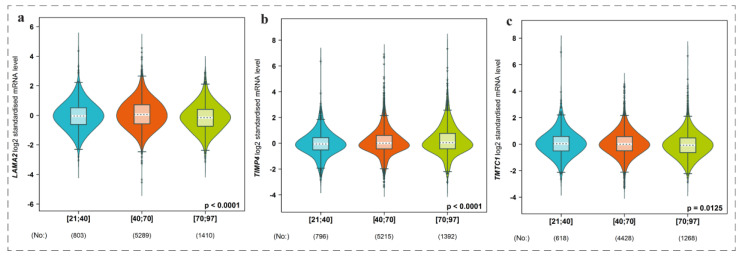
Violin plot showing gene expression among groups of patients categorized according to age (**a**–**c**). We found that the genes namely (**a**) *LAMA2*, (**b**) *TMTC1*, and (**c**) *TIMP4* were significantly expressed among different age groups of patients, i.e., lower 21 age to higher 97 age groups as indicated by the violin plots.

**Figure 7:**
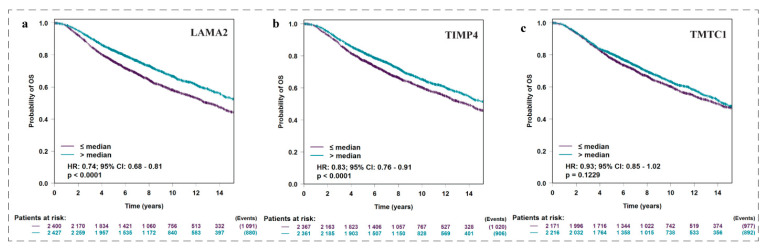
Asociation of genes with breast cancer occurrence (**a**–**c**). (**a**) LAMA2 and (**b**) TIMP4 were found significantly associated and (**c**) TMTC1 gene was found less correlated with breast cancer occurrence.

We have used bc-GenExMiner web dependent tool to compare expression level of genes among groups of patients with respect to age factor. We found that the genes namely *LAMA2*, *TMTC1*, and *TIMP4* were significantly expressed among different age groups of patients, i.e., lower 21 age to higher 97 age groups as indicated by the violin plots (Figure 6). Moreover, we further deeply investigated the role of *LAMA2*, *TMTC1* and *TIMP4* genes in breast cancer prognosis. We found that *LAMA2* and *TIMP4* were significantly associated and *TMTC1* gene was less correlated with breast cancer occurrence (Figure 7).

### 3.9. Pan-Cancer View of LAMA2, TIMP4, and TMTC1 Expression Level Using UALCAN Analysis

**Figure 8:**
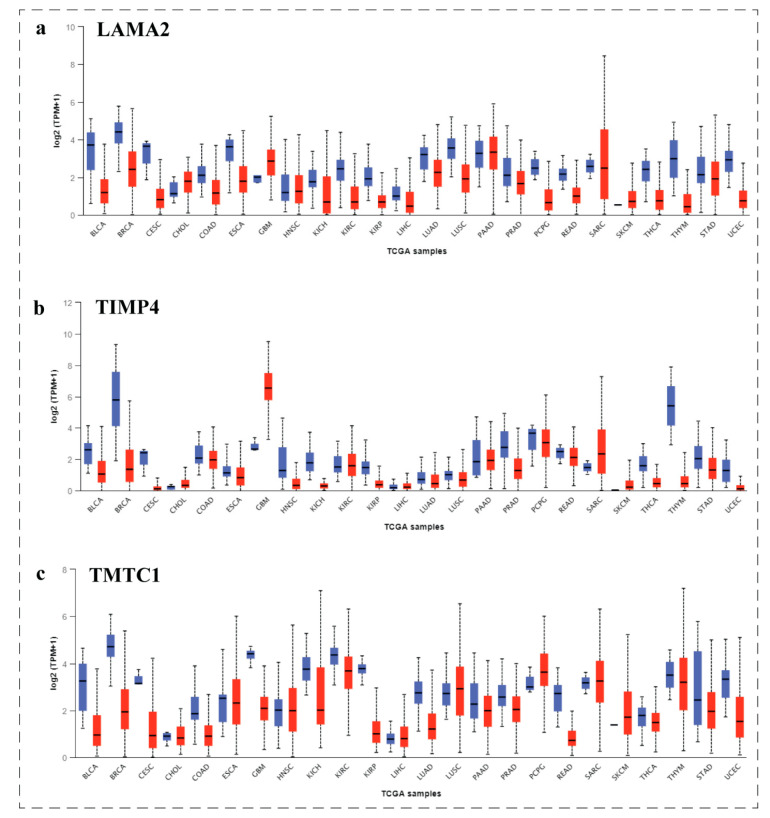
Pan-cancer view of *LAMA2*, *TIMP4*, and *TMTC1* expression level (**a**–**c**). We found that the expression level of (**a**) LAMA2, (**b**) TIMP4, and (**c**) TMTC1 was higher in all TCGA tumors.

UALCAN is a comprehensive, user-friendly, and interactive web resource for analyzing cancer OMICS data. UALCAN enables researchers to access Level 3 RNA-seq data from The Cancer Genome Atlas (TCGA) and perform gene expression and survival analysis on about 20,500 protein-coding genes in 33 different tumor types [8]. Expression levels of *LAMA2*, *TIMP4*, and *TMTC1* across TCGA tumours are shown in (Figure 8). We found that the expression level of *LAMA2*, *TIMP4*, and *TMTC1* was higher in all TCGA tumours.

### 3.10. Effect of Expression Levels of LAMA2, TIMP4, and TMTC1 on the Survival of Breast Cancer Patient Oncolnc Analysis 

**Figure 9:**
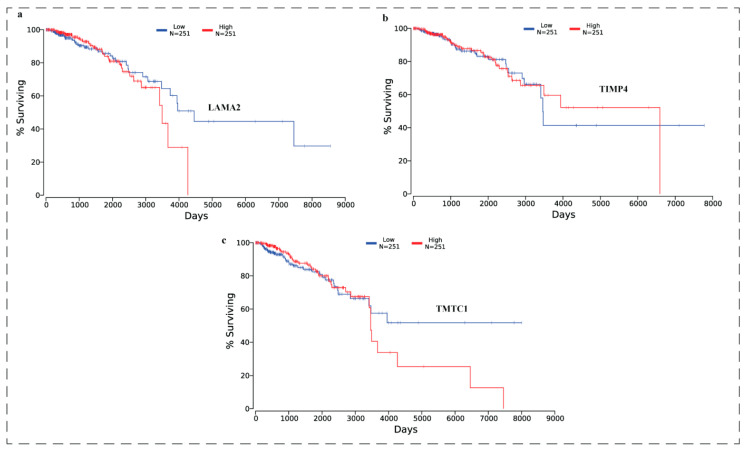
Analysis of the prognostic value of three differentially expressed genes in breast cancer patients using The Cancer Genome Atlas data. All the three genes (**a**) LAMA2, (**b**) TIMP4, and (**c**) TMTC1were found significantly (*p* < 0.05) associated with poor survival.

OncoLnc was used to analyse the effect of expression levels of *LAMA2*, *TIMP4*, and *TMTC1* on the survival of breast cancer patients. Results are presented in the (Figure 9). Results showed that all the three genes were significantly (*p* < 0.05) associated with poor survival.

### 3.11. Expression of Key DEGs in Human Breast Cancer Cell Lines Using qRT-PCR

**Figure 10:**
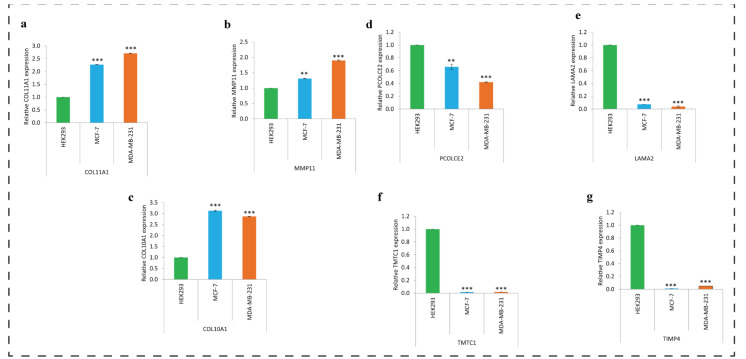
Validation of key regulatory genes in human breast cancer cell lines using qRT-PCR. In the figure, (**a**–**c**) presents up-regulated and (**d**–**g**) presents down-regulated genes, ** *p* < 0.01, *** *p* < 0.001.

The qRT-PCR analysis showed similar type of expression profiles of key regulatory genes as revealed by the bioinformatics analysis except the two genes namely ADAMTS5 and RSPO3. Results are presented as (Figure 10a–g). The genes namely COL11A1, MMP11 and COL10A1 (Figure 10a–c) were found up-regulated and PCOLCE2, LAMA2, TMTC1, and TIMP4 (Figure 10d–g) were found down-regulated in breast cancer cell lines also. We could not determine the expression of two genes namely ADAMTS5 and RSPO3.

## 4. Discussion

Breast cancer associated higher mortality rate reflect the need of identification and discovering new biomarkers, therapeutic molecules, and molecular therapeutic targets which will pave for the development of early diagnosis and effective treatment. Identification of imperative gene targets associated with the cancer phenotypes is essential for the development of successful therapy. Currently, at larger scale in-silico techniques are implemented to discover the key regulatory genes. Analysis of gene expression profiles from different databases provides a plinth to quantify and differentiate the gene expression level between normal and tumor samples. Hence, the main aim of this proposed investigation was in-silico identification and in-vitro validation of key regulatory genes associated with breast cancer phenotypes. We conducted an integrative bioinformatics analysis by comparing the normal and breast cancer samples from four transcriptomic datasets. Initially, 161 common differentially expressed genes were extracted from all the four datasets. Among these differentially expressed genes, 44 genes were up-regulated, and 117 genes were found down-regulated. Then we constructed the PPI network, which showed 449 nodes and 18214 edges. To characterize the modular nature, attributes, and organizing principle of the hierarchical network, the activities of the created network were defined at various levels of hierarchy. The modules from the native network along with sub-modules from modules at each level of organization were identified until only motifs remained i.e., unbreakable part of the network. We found that our PPI network is organized hierarchically through seven different levels. We traced these modules to find out the leading hubs or key regulators. Only those that regulate the network from top to bottom (where the PPI network cannot be further divided into sub-community and form motif) are considered as important leading hubs or key regulators. We identified nine key regulators namely *PCOLCE2* (Procollagen C-Endopeptidase Enhancer 2), *LAMA2* (Laminin Subunit Alpha 2), *TMTC1* (transmembrane O-mannosyltransferase targeting cadherins 1), *ADAMTS5* (ADAM Metallopeptidase with Thrombospondin Type 1 Motif 5), *TIMP4* (TIMP-Metallopeptidase Inhibitor 4), *RSPO3* (R-Spondin 3), *COL11A1* (Collagen Type XI Alpha 1 Chain), *MMP11* (matrix metalloproteinase-11), and *COL10A1* (Collagen Type X Alpha 1 Chain). These nine genes were termed as network’s key regulators or organizers, as reflected by the PPI network analysis. Among these key genes *COL11A1*, *MMP11* and *COL10A1* were highly expressed and *PCOLCE2*, *LAMA2*, *TMTC1*, *ADAMTS5*, *TIMP4*, and *RSPO3* were having lower expression levels in breast cancer samples. Similar expression profile of seven among nine genes (except *ADAMTS5* and *RSPO3*) was validated in MCF-7 and MDA-MB-231 human breast cancer cell lines. These validation observations reflect that these key genes may pave the way for effective therapeutics of breast cancer. *COL11A1* has been reported markedly associated with head and neck, oral cavity/pharynx, breast, oesophagus, lung, colon, stomach, ovary, and pancreas cancers [39,40]. It has been found well correlated with adverse clinical outcomes in breast cancer [41], recurrence in glioblastoma and ovarian cancer [42,43], and poor survival of kidney and ovarian cancer patients [37,44]. Distinct roles of *MMP11* in cancer development, progression and therapeutics have been reported (reviewed in [45]). *MMP11* has been suggested as a novel target antigen for cancer immunotherapy [46]. High *MMP11* expression has been found associated with poor survival of breast cancer patients [47]. Reports highlight that the expression of *COL10A1* is markedly increased in colon, esophagus, and breast cancer and contributes to cell proliferation, migration, invasion and tumor vasculature [48,49,50]. Previous studies suggest that expression pattern of *COL10A1* might act as a potential diagnostic predictor for early breast cancer [51]. *PCOLCE2* has been implicated in the colorectal cancer [5] and gastric cancer [52], however, the expression pattern of *PCOLCE2* is poorly understood. Decreased expression of *LAMA2* has been reported in various cancers [53]. *LAMA2* has also been found well correlated with tumor sites and to predict poor survival in pancreatic cancer [54]. *TMTC1* has been also found associated with gastric cancer and has been suggested to act as serve as predictive biomarker for gastric cancer treatment [55]. It is well documented that *ADAMTS5* shows tumor type specific functions. *ADAMTS5* has been reported to act as a tumor suppressor gene in breast cancer [56] and hepatocellular carcinoma [57]. *TIMP4* has been found associated with breast cancer to modulate the ER-α Signalling in MCF7 Breast Cancer Cells [58]. *RSPO3* has been implicated in ovarian cancer and has been suggested as candidate marker to predict ovarian cancer aggressiveness [59]. 

Next, we mapped all these key genes to GO analysis. We found that these key regulators were involved in several GO biological processes namely extracellular structure organization, external encapsulating structure organization, extracellular matrix organization, extracellular matrix disassembly, cellular component disassembly, collagen fibril organization, and supramolecular fibre organization. GO Cellular Components associated with key genes include endoplasmic reticulum lumen, intracellular organelle lumen, collagen-containing extracellular matrix, basement membrane, and Golgi lumen. GO Molecular Function analysis showed the key genes were associated with the molecular functions namely metalloendopeptidase activity, metallopeptidase activity, metalloendopeptidase inhibitor activity, and endopeptidase activity. These key regulators were enriched in protein digestion and absorption and viral myocarditis according to the KEGG pathway analysis. All these findings reflect that these key genes are associated with crucial biological functioning and hence can be implicated for the therapeutics of cancer. Further, we found that the genes namely *LAMA2*, *TMTC1*, and *TIMP4* were significantly expressed among different age groups of patients. *LAMA2* and *TIMP4* were found significantly associated and *TMTC1* gene was found less correlated with breast cancer occurrence. We found that the expression level of *LAMA2*, *TIMP4*, and *TMTC1* was higher in all TCGA tumours and significantly associated with poor survival.

While bioinformatics-based transcript profiling is a powerful tool for characterizing the molecular features of breast cancer subtypes, there are limitations associated to this approach. Bioinformatics-based gene expression profiling may reveal distinct gene expression patterns that are associated with different subtypes of breast cancer such as in hormone receptor-positive breast cancer, HER2-positive breast cancer, and Triple-negative breast cancer. Generally, in hormone receptor-positive breast cancer, there is overexpression of estrogen receptor (ER) and/or progesterone receptor (PR) genes, increased expression of genes involved in cell proliferation and cell cycle regulation, such as *Ki-67* and *Cyclin D1*, high expression of genes involved in estrogen signaling, such as *GATA3* and *FOXA1*, and Low expression of genes involved in immune response and inflammation, such as *TNF* and *IL6*. In HER2-positive breast cancer, there is overexpression of the *HER2* gene and other genes in the HER2 signaling pathway, increased expression of genes involved in cell proliferation and survival, such as *MYC* and *BCL2*, high expression of genes involved in DNA repair and genomic stability, such as *BRCA1* and *BRCA2*, and low expression of genes involved in immune response and inflammation, such as *TNF* and *IL6*. In triple-negative breast cancer, there is low expression of hormone receptors (*ER* and *PR*) and *HER2* gene, increased expression of genes involved in cell cycle regulation, such as *Cyclin B1* and *CDC20*, high expression of genes involved in DNA damage repair, such as *RAD51* and *BRCA1*, and high expression of genes involved in immune response and inflammation, such as *IFNγ* and *TNFα*. It’s important to note that these transcript profile characteristics in bioinformatics-based gene expression profiling may not be absolute and can vary among individual tumors within a given subtype. In addition, bioinformatics analysis based identified genes could be also general prognostic markers for other types of tumors, but not specific to breast tumors only. Nonetheless, the bioinformatics bases gene expression profiling has helped to identify potential therapeutic targets and develop personalized treatment strategies for many diseases.

## 5. Conclusions

In conclusion, our study identified nine key regulators, of which *COL11A1*, *MMP11* and *COL10A1* were up regulated and *PCOLCE2*, *LAMA2*, *TMTC1*, *ADAMTS5*, *TIMP4* and *RSPO3* were down regulated in breast cancer samples as compared to control samples. Expression level of *LAMA2*, *TIMP4*, and *TMTC1* was higher in all different stages of TCGA breast cancer samples and significantly expressed among different age groups of patients (younger to older age group). *LAMA2* and *TIMP4* were significantly associated and *TMTC1* gene was less correlated with breast cancer occurrence. Survival analysis of the genes showed significant association of *LAMA2*, *TIMP4*, and *TMTC1* were significantly associated with poor survival.

## Figures and Tables

**Table 1:** Detailed information of datasets used to extract differentially expressed genes.

Datasets	Total Samples	Normal (No. of Samples and Age)		Disease (No. of Samples and Age)		Up Regulated	Down Regulated	Country	Platform	Author
GSE29044	43	5 (Age 20–35 years)	7 (Age > 55 years)	6 (Age 20–35 years)	25 (Age > 55 years)	490	716	Saudi Arabia	GPL570	Colak D
GSE89116	33	4 (Age upto 35 years)	5 (Age upto 80 years)	11 (Age upto 38 years)	13 (Age upto 80 years)	186	542	India	GPL6947	Malvia S
GSE109169	50	5 (Age upto 40 years)	20 (Age > 40 years)	5 (Age upto 40 years)	20 (Age > 40 years)	219	358	Taiwan	GPL570	Chang JW
GSE42568	121	17 (Age not mentioned)		104 (Age 31–89 years)		896	1202	Ireland	GPL570	Clarke C

**Table 2:** All common differentially expressed genes (DEGs).

*IGF1, LIFR, SFRP1, TSHZ2, SPTBN1, TMEM47, MME, NTRK2, PLAGL1, DMD, MAMDC2, LPL, HLF, PROS1, FABP4, ABCA9, ADAMTS5, RBMS3, APOLD1, SPRY2, ANGPTL1, EBF1, ITM2A, MEOX2, CAV1, TGFBR3, OGN, ANKRD29, CRYAB, CLIP4, CD36, EDNRB, CAV2, FAM13A, SYNM, ADH1B, FHL1, CDO1, TFPI, GPAM, CHRDL1, ABCA8, LYVE1, CACHD1, ITIH5, PLSCR4, SORBS1, LARP6, RUNX1T1, GPC3, PCDH18, AKAP12, PDK4, ACACB, ECM2, SPRY1, PAMR1, CXCL12, ADIPOQ, EMCN, FGF2, ATP1A2, ADH1C, PCOLCE2, ABCA6, SIK2, PCDH9, TMEM100, ARHGAP20, CHL1, MAOA, MTURN, LEP, SLIT3, IGSF10, GSTM5, VIT, RBP7, GPD1, CIDEC, MYZAP, RSPO3, FAXDC2, CFD, FBLN5, LDB2, GNG11, TEK, RBP4, FAM107A, ENPP2, PLIN1, CPED1, TIMP4, GSN, LMOD1, ALDH1A1, LAMA2, GPX3, AOC3, MT1M, JAM2, FXYD1, PPARG, APOD, DPT, G0S2, SH3BGRL2, VGLL3, DLC1, PCK1, C7, PDGFD, ANK2, GHR, TMTC1, CCDC3, PRC1, RRM2, TPX2, CDKN3, DTL, KIF4A, FAM83D, UBE2T, NUF2, MMP11, COL10A1, CCNB1, CDK1, DEPDC1, AURKA, CENPF, KIF23, KIF11, ANLN, FN1, EZH2, TRIP13, PBK, DLGAP5, UHRF1, TK1, CCNB2, MELK, CENPU, ATAD2, HMMR, ECT2, NUSAP1, ASPM, FANCI, SQLE, TOP2A, KIF20A, RACGAP1, GJB2, TLCD1, COL11A1, LRRC15, CKS2*

**Table 3:** List of key up- and down-regulated genes with LogFC, *p*-value, and Adj. *p*. Value.

Genes	LogFC	*p* Value	Adj. *p* Value
*PCOLCE2*	−4.45	2.44 × 10^−11^	1.67 × 10^−7^
*LAMA2*	−1.89	3.21 × 10^−4^	1.26 × 10^−2^
*TMTC1*	−2.49	2.97 × 10^−3^	4.98 × 10^−2^
*ADAMTS5*	−2.31	1.80 × 10^−4^	9.13 × 10^−3^
*TIMP4*	−2.54	1.40 × 10^−4^	7.60 × 10^−3^
*RSPO3*	−2.58	5.07 × 10^−5^	4.03 × 10^−3^
*COL11A1*	5.29	9.91 × 10^−14^	3.60 × 10^−12^
*MMP11*	4.62	4.13 × 10^−6^	8.01 × 10^−4^
*COL10A1*	3.49	6.29 × 10^−13^	2.01 × 10^−11^

## Data Availability

All related data are available within the article.

## Supplementary Materials

The following supporting information can be downloaded at: https://www.mdpi.com/article/10.3390/biomedicines11051271/s1, Figure S1: A pictorial flow chart of network breakdown.: Figure S2: The behaviors of topological properties: Betweenness (CB(k)), closeness (CC(k)), degree distributions (P(k)) at different levels of organization.; Table S1: List of qRT-PCR primers of key genes.; Table S2: GO Biological Process; Table S3: GO Cellular Component; Table S4: GO Molecular Function; Table S5: KEGG pathways.
