# Supplementary material for: Identification of New Key Genes and Their Association with Breast Cancer Occurrence and Poor Survival Using In Silico and In Vitro Methods
Source: Biomedicines. 2023 Apr 25;11(5):1271. doi: 10.3390/biomedicines11051271 (PMC10216146; doi:10.3390/biomedicines11051271)
Supplement: Supplementary file 1 [file biomedicines-11-01271-s001.zip › biomedicines-2221890-supplementary.pdf]

Legends-

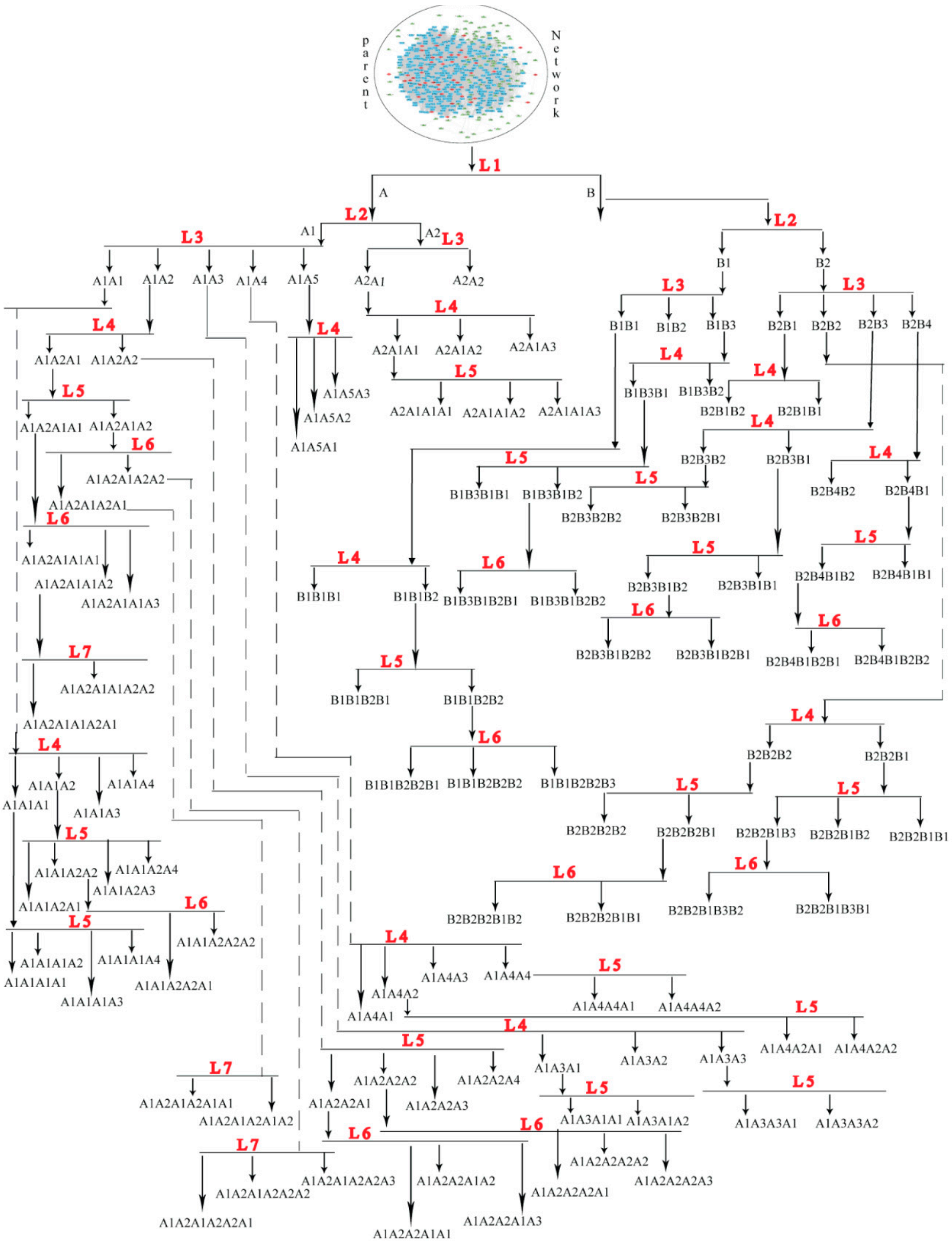

Figure S1. A pictorial flow chart of network breakdown.

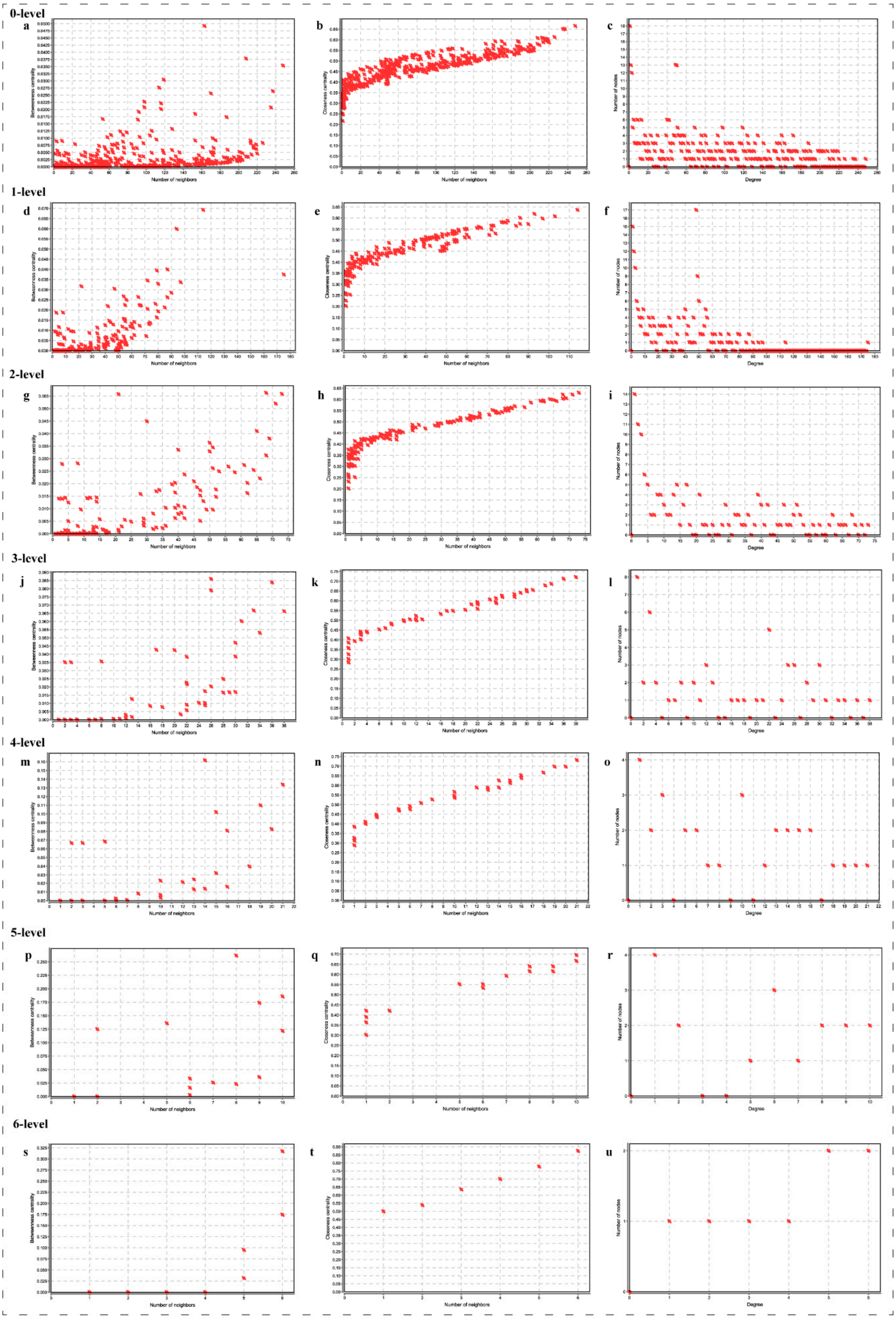

Figure S2. The behaviours of topological properties: betweenness ($C_B(k)$), closeness ($C_C(k)$), and degree distributions ($P(k)$) at different levels of organization.

**Table S1.** List of qRT-PCR primers of key genes.		
Genes	Forward	Reverse
PCOLCE2	CCTTCCGGCTCTTTTAAAAC	TATCTCGCTCCACATCAAAC
LAMA2	CTGACAGTTGAACAGCCTAT	CCATATGCAGCCTTCAAAAG
TMTC1	GTGTGGTCCATGATGAGATT	CAGAAAGATGGTGGCTAAGT
ADAMTS5	CCATCCTTACCAGCATTGAT	TAGGTCCAGCAAACAGTTAC
TIMP4	GGCTCAGCATTATGTCTGTA	GCTTGATCTTCAGGACTCTT
RSPO3	AGTCAGCACTGTACACTAGA	CATGTCCATTTGTGGTCCTA
COL11A1	AGTATGGGGAAGCAGAGTAT	TAGGAGCTTCTGTCTGGTAA
MMP11	TCCCGAGAAGAACAAGATCT	AGGGTCAAACTTCCAGTAGA
COL10A1	GGAGGTAGGCTGAAAAGAAT	GCAGGACTTCTTTGGTGATA

**Table S2.** GO Biological Process.				
**S. No.**	**Term**	**GO**	***p*-value**	**Genes**
1.	Extracellular structure organization	(GO:0043062)	1.71E-08	*ADAMTS5, MMP11, LAMA2, COL11A1, COL10A1*
2.	External encapsulating structure organization	(GO:0045229)	1.75E-08	*ADAMTS5, MMP11, LAMA2, COL11A1, COL10A1*
3.	Extracellular matrix organization	(GO:0030198)	8.81E-08	*ADAMTS5, MMP11, LAMA2, COL11A1, COL10A1*
4.	Extracellular matrix disassembly	(GO:0022617)	3.80E-04	*ADAMTS5, MMP11*
5.	Cellular component disassembly	(GO:0022411)	3.80E-04	*ADAMTS5, MMP11*
6.	Collagen fibril organization	(GO:0030199)	6.91E-04	*COL11A1, COL10A1*
7.	Positive regulation of Wnt signalling pathway, planar cell polarity pathway	(GO:2000096)	0.002697256	*RSPO3*

8.	Negative regulation of membrane protein ectodomain proteolysis	(GO:0051045)	0.003146172	*TIMP4*
9.	Regulation of Wnt signalling pathway, planar cell polarity pathway	(GO:2000095)	0.004043466	*RSPO3*
10.	Detection of mechanical stimulus involved in sensory perception of sound	(GO:0050910)	0.004043466	*COL11A1*
11.	Positive regulation of non-canonical Wnt signalling pathway	(GO:2000052)	0.004940043	*RSPO3*
12.	Myoblast fusion	(GO:0007520)	0.006731046	*ADAMTS5*
13.	Regulation of non-canonical Wnt signalling pathway	(GO:2000050)	0.006731046	*RSPO3*
14.	detection of mechanical stimulus involved in sensory perception	(GO:0050974)	0.006731046	*COL11A1*
15.	Protein O-linked mannosylation	(GO:0035269)	0.008072419	*TMTC1*
16.	myotube differentiation	(GO:0014902)	0.009412182	*ADAMTS5*
17.	Protein mannosylation	(GO:0035268)	0.009858413	*TMTC1*
18.	regulation of membrane protein ectodomain proteolysis	(GO:0051043)	0.009858413	*TIMP4*
19.	Supramolecular fibre organization	(GO:0097435)	0.010190894	*COL11A1, COL10A1*
20.	Maintenance of blood–brain barrier	(GO:0035633)	0.013421829	*LAMA2*
21.	Endodermal cell differentiation	(GO:0035987)	0.014310901	*COL11A1*
22.	Endoderm formation	(GO:0001706)	0.016086908	*COL11A1*
23.	Negative regulation of protein catabolic process	(GO:0042177)	0.018745587	*TIMP4*
24.	Negative regulation of proteolysis	(GO:0045861)	0.019188079	*TIMP4*
25.	Negative regulation of cold-induced thermogenesis	(GO:0120163)	0.020956273	*ADAMTS5*
26.	Sprouting angiogenesis	(GO:0002040)	0.023162532	*RSPO3*
27.	Negative regulation of metabolic process	(GO:0009892)	0.024924356	*ADAMTS5*

28.	Muscle organ development	(GO:0007517)	0.025804208	*LAMA2*
29.	Negative regulation of peptidase activity	(GO:0010466)	0.028000753	*TIMP4*
30.	Negative regulation of endopeptidase activity	(GO:0010951)	0.028000753	*TIMP4*
31.	Negative regulation of cellular catabolic process	(GO:0031330)	0.030630795	*TIMP4*
32.	Regulation of endopeptidase activity	(GO:0052548)	0.031506069	*TIMP4*
33.	Sensory perception of mechanical stimulus	(GO:0050954)	0.038917584	*COL11A1*
34.	Sensory perception of sound	(GO:0007605)	0.040220252	*COL11A1*
35.	Visual perception	(GO:0007601)	0.041087826	*COL11A1*
36.	Sensory perception of light stimulus	(GO:0050953)	0.041954702	*COL11A1*
37.	Protein O-linked glycosylation	(GO:0006493)	0.044983286	*TMTC1*
38.	Regulation of Wnt signalling pathway	(GO:0030111)	0.048864674	*RSPO3*
39.	Response to cytokine	(GO:0034097)	0.065522489	*TIMP4*
40.	Positive regulation of Wnt signalling pathway	(GO:0030177)	0.066793052	*RSPO3*
41.	Skeletal system development	(GO:0001501)	0.068907244	*COL10A1*
42.	Regulation of signal transduction	(GO:0009966)	0.085668109	*RSPO3*
43.	Axon guidance	(GO:0007411)	0.087744244	*LAMA2*
44.	Negative regulation of multicellular organismal process	(GO:0051241)	0.092296998	*ADAMTS5*
45.	Axonogenesis	(GO:0007409)	0.102977865	*LAMA2*
46.	Positive regulation of signal transduction	(GO:0009967)	0.107869705	*RSPO3*

**Table S3.** GO Cellular Component.				
**S. No.**	**Term**	**GO**	**P-value**	**Genes**
1.	Endoplasmic reticulum lumen	(GO:0005788)	2.26E-04	ADAMTS5, COL11A1, COL10A1
2.	Intracellular organelle lumen	(GO:0070013)	3.41E-04	ADAMTS5, MMP11, COL11A1, COL10A1
3.	Collagen-containing extracellular matrix	(GO:0062023)	5.25E-04	LAMA2, COL11A1, COL10A1
4.	Basement membrane	(GO:0005604)	0.023162532	LAMA2
5.	Golgi lumen	(GO:0005796)	0.044118846	MMP11

**Table S4.** GO Molecular Function				
**S. No.**	**Term**	**GO**	***p*-value**	**Genes**
1.	Metalloendopeptidase activity	(GO:0004222)	5.87E-04	*ADAMTS5, MMP11*
2.	Metallopeptidase activity	(GO:0008237)	0.001271025	*ADAMTS5, MMP11*
3.	Metalloendopeptidase inhibitor activity	(GO:0008191)	0.006283564	*TIMP4*
4.	Endopeptidase activity	(GO:0004175)	0.008274373	*ADAMTS5, MMP11*
5.	Mannosyltransferase activity	(GO:0000030)	0.009858413	*TMTC1*
6.	Peptidase activator activity	(GO:0016504)	0.009858413	*PCOLCE2*
7.	Endopeptidase inhibitor activity	(GO:0004866)	0.050585229	*TIMP4*
8.	Protease binding	(GO:0002020)	0.05187383	*TIMP4*
9.	Hexosyl transferase activity	(GO:0016758)	0.052303018	*TMTC1*

**Table S5.** KEGG pathways.			
**S. No.**	**Term**	***p*-value**	**Genes**
1.	Protein digestion and absorption	9.24E-04	COL11A1, COL10A1
2.	Viral myocarditis	0.026683	LAMA2
3.	Arrhythmogenic right ventricular cardiomyopathy	0.034128	LAMA2
4.	ECM-receptor interaction	0.038918	LAMA2
5.	Hypertrophic cardiomyopathy	0.039786	LAMA2
6.	Small cell lung cancer	0.040654	LAMA2
7.	Dilated cardiomyopathy	0.042388	LAMA2
8.	Amoebiasis	0.044983	LAMA2
9.	Toxoplasmosis	0.049295	LAMA2
10.	Wnt signalling pathway	0.072281	RSPO3
11.	Focal adhesion	0.086914	LAMA2
12.	Human papillomavirus infection	0.139487	LAMA2
13.	PI3K-Akt signalling pathway	0.148502	LAMA2
14.	Pathways in cancer	0.215123	LAMA2
